# Supplementary material for: Immune Contexture of MMR-Proficient Primary Colorectal Cancer and Matched Liver and Lung Metastases
Source: Cancers (Basel). 2021 Mar 26;13(7):1530. doi: 10.3390/cancers13071530 (PMC8037224; doi:10.3390/cancers13071530)
Supplement: Supplementary file 1 [file cancers-13-01530-s001.pdf]

cancers

MDPI

Immune Contexture of MMR-Proficient Primary Colorectal Cancer and Matched Liver and Lung Metastases

Maarit Ahtiainen, Hanna Elomaa, Juha P. Väyrynen, Erkki-Ville Wirta, Teijo Kuopio, Olli Helminen, Toni T. Seppälä, Ilmo Kellokumpu and Jukka-Pekka Mecklin

Table S1. Comparison of the categorized immune variables between primary tumours and matched metastases.

	Liver-only			Both liver and lung			Lung-only		
	Primary tumours *n* = 72	Metastases *n* = 83	*P*-value	Primary tumours *n* = 18	Metastases *n* = 53	*P*-value	Primary tumours *n* = 23	Metasta-ses *n* = 28	*P*-value
**PD-L1^{TC}**									
low	71 (99)	80 (98)	0.638	17 (100)	51 (96)	0.416	23 (100)	27 (96)	0.360
high	1 (1)	2 (2)		0 (0)	2 (4)		0 (0)	1 (4)	
**PD-L1^{IC}**									
low	42 (52)	39 (48)	0.158	13 (77)	20 (38)	**0.005**	14 (61)	3 (11)	**< 0.001**
high	30 (48)	43 (52)		4 (23)	33 (62)		9 (39)	25 (89)	
**Immune cell score**									
Low (0-2)	47 (68)	40 (50)	**0.025**	12 (67)	26 (54)	0.360	14 (64)	12 (46)	0.351
High (3-4)	22 (32)	40 (50)		6 (33)	22 (46)		8 (36)	14 (54)	
**Im-munopro-file**									
0	27 (39)	19 (24)	**0.045**	6 (35)	12 (24)	0.128	8 (36)	1 (4)	**0.009**
1	11 (16)	23 (29)		8 (47)	14 (29)		6 (27)	8 (33)	
2	19 (28)	16 (20)		2 (12)	6 (12)		5 (23)	3 (13)	
3	12 (17)	22 (27)		1 (6)	17 (35)		3 (14)	12 (50)	

Abbreviations: PD-1, programmed cell death protein 1; PD-L1, programmed death ligand 1; TC, tumour cell; IM, invasive margin; IC, immune cell. Because of indeterminable samples either in CD3, CD8, PD-1 or PD-L1, sample numbers in Immune cell score and Immunoprofile do not completely correspond to the total sample number.
